# Experiences of group antenatal care in the context of the NHS in England: what are the mechanisms by which it functions in this context?

**DOI:** 10.3389/fgwh.2025.1625785

**Published:** 2025-10-24

**Authors:** Christine McCourt, Anita Mehay, Octavia Wiseman, Jalana Lazar, Ruth Ajayi, Thomas Hamborg, Vivian Holmes, Rachael Maree Hunter, Ekaterina Mishareva, Pearl Safo Sobre, Meg Wiggins, Angela Harden, Cathy Salisbury, Bethan Hatherall

**Affiliations:** 1 Centre for Maternal and Child Health Research, School of Health Sciences, City, University of London, London, United Kingdom; 2 City St George's, University of London, London, United Kingdom; 3 PPIE Co-Investigator, London, United Kingdom

**Keywords:** group antenatal care, pregnancy circles, Centering Pregnancy, experience, mechanisms, empowerment, continuity

## Abstract

**Introduction:**

Group antenatal care is a model where care is provided in groups of around 6–12 women/birthing people, integrating healthcare with information and learning in a participatory approach. There is international evidence of improved care experiences and outcomes; however, the approach (here called Pregnancy Circles) had not been trialled in the United Kingdom in the context of a universal health system with midwife-led care. We aimed to understand the experience of care and any mechanisms by which group care functions for the different people involved.

**Method:**

This study comprised a qualitative process evaluation nested within a randomised controlled trial. The mixed qualitative methods used in this study included observations of care, interviews with participants, survey open-text responses and written feedback, and a review of relevant documents. Inductive thematic analysis was conducted using a framework of theorised mechanisms based on a realist review. The trial’s clinical and psychosocial outcomes and lessons for implementation are reported elsewhere.

**Results:**

We found a high level of concordance with the framework of mechanisms derived from the literature. The key mechanisms were social support and community building, a critical pedagogy (combining peer learning, an interactive and participatory approach, and health education), satisfaction and engagement with care, and the health professionals’ satisfaction and development. Building on these, the empowerment of participants and midwives formed an overarching mechanism. Relational continuity and time for care were the key underpinning components.

**Discussion:**

Pregnancy Circles address key deficits in contemporary maternity care, including the lack of time and relational or informational continuity of care, the lack of informed choice, and loss of opportunities to enhance empowerment through health knowledge, social support, and confidence in caring for one's own health, in decision-making, and in seeking support. Importantly, midwives felt that facilitating group care enhanced their professional satisfaction and development and collaboration across boundaries, features associated with service safety and resilience. Fidelity in terms of the midwives' skills and confidence in using a facilitative approach was important and was underpinned by continuity. Midwives' and women's empowerment were found to be mutually supportive rather than in tension. Scaling up Pregnancy Circles as a standard care option in the National Health Service may support positive care experiences; however, further research is needed to monitor the longer-term impact and service and public health implications.

## Introduction

Pregnancy Circles (PC) is a model of group antenatal care adapted for the United Kingdom’s National Health Service (NHS) setting that is aligned with the Centering Pregnancy model introduced in the United States. Group antenatal care involves providing the usual schedule of antenatal care (ANC), with 90–120 min per visit (rather than the typical 15–30 min), to a group of around 6–12 women with births due around the same time, rather than individually, which is facilitated by two professionals—midwives in the case of the United Kingdom. Depending on the context, partners may be included in all or in selected sessions; at our study sites, this was decided by the women in the group during their first session. Although satisfaction with antenatal care in the United Kingdom is generally high, there is evidence of inequity in access and quality of care for Black and South Asian heritage women and those who are more socioeconomically disadvantaged (1), levels of informed choice and continuity of carer are limited (2–4), and antenatal education is not always accessible or of a high quality (5). Group care aims to enable a more active and interactive approach to learning, with a facilitative rather than didactic approach, engaging pregnant women/birthing people more fully in their care, including conducting their own routine health checks, such as blood pressure monitoring, within the group space. It also seeks to enable a higher level of social support from peers and from midwives. Previous studies have identified a range of potential benefits, including improved uptake and experience of care (6, 7), reduced preterm birth or low birthweight among women in more vulnerable situations (8), and increased breastfeeding rates (9). A small-scale pilot study of Centering Pregnancy in the United Kingdom reported positive responses among women and midwives but was not continued by the service (10).

**Figure 1:**
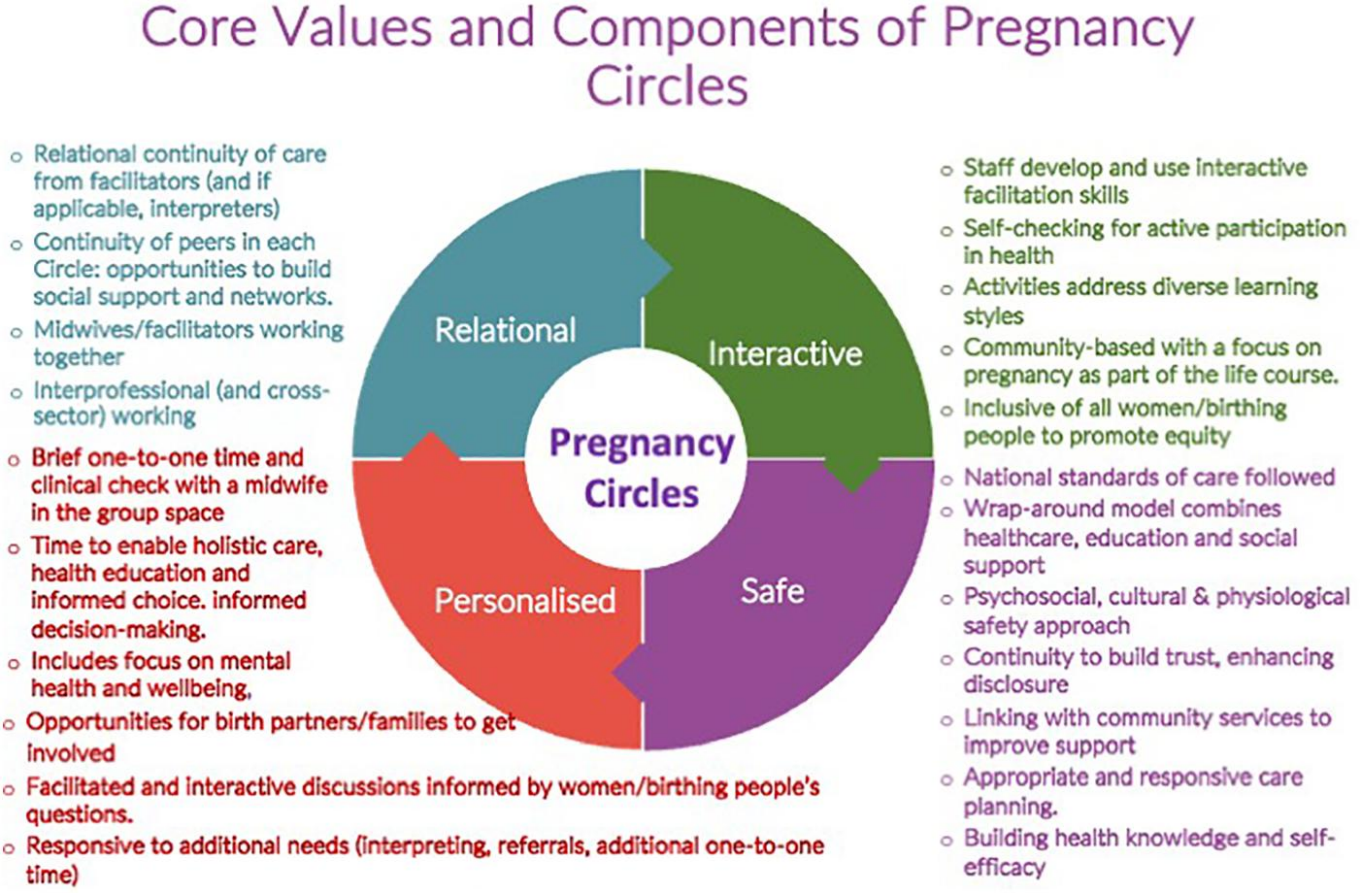
Core values and components model [reproduced from Wiseman et al. (17)].

The Pregnancy Circles trial grew from a community-based co-design process, exploring ways to improve equity in access and quality of antenatal care. A feasibility study identified positive experiences among both women and midwives (11, 12). A core theme of “Better Together” (being in the group) captured the experience of social support within a safe group space that also provided clinical care (12). Midwife participants valued a more relational approach to care, which felt like “real midwifery” (12). The feasibility work established that, despite the reservations of some service managers, the approach would be acceptable to women from diverse social and ethnic backgrounds, and that diversity within groups, including parity, obstetric risk, and social factors, was preferred (11). A pilot RCT indicated feasibility (13, 14), including the feasibility of including women with limited English proficiency with interpreter support (15). An individually randomised multicentre controlled trial with integral process and economic evaluations was conducted from 2018 to 2024 (including a 26-month pause relating to the COVID-19 pandemic) across 14 NHS Trusts in England (16). The key values of the Pregnancy Circles model were identified through this process to be the following: relational, interactive, personalised, and safe (Figure 1).

A realist review conducted alongside this work to understand theories of effect within the existing research and professional literature identified a range of candidate mechanisms by which group antenatal care may enhance care experiences and/or outcomes across different settings (18).

In this article, we report findings from the process evaluation that explore and identify the mechanisms by which group antenatal care functioned, or did not, to enhance care experience and outcomes within an NHS setting at an early implementation stage. In this approach, mechanisms are conceptualised as the means by which a programme or intervention works “through changing the reasoning and responses of participants to bring about a set of intended outcomes” (19). While components of an intervention may be standardised, they may be adapted in planned or unplanned ways and mechanisms can vary, shaped by contexts and actions. Findings related to implementation facilitators and barriers, clinical and psychosocial outcomes, and cost-effectiveness are reported separately (17).

## Methods

We conducted a qualitative process evaluation informed by realist evaluation principles (19) to examine the providers’ and service-users’ experiences of care and the presence or absence of treatment effects and to identify any unanticipated or unintended consequences. This included a focus on the implementation context and process and how the model was implemented in practice, acknowledging that practices and experiences may vary across different settings and participants. Implementation-related findings are reported elsewhere (17).

Data collection was mainly conducted at three “case study” sites, selected from within the 14 trial sites for variation of context, with additional data collection at 8 of the other trial sites where needed to address any gaps or questions generated during the process. Group care is a complex intervention requiring adaptation at the organisational and professional levels, including adaptations to a more facilitative and interactive way of working, co-working with other professionals, and sharing experience with other pregnant women/birthing people and their birth partners. Therefore, variation is expected in how the care functions in different contexts and the responses of different participants. Mechanisms are, therefore, a combination of the intervention itself, how it is implemented in different contexts, and how participants interact with it (18, 19). In earlier stages of the work, we developed a logic model to represent the research team's initial programme theory and a core values and components model (16). This will contribute to a final programme theory, incorporating both the trial’s and process evaluation’s findings.

A mix of qualitative methods was used to develop a rounded understanding of how the care was implemented, provided, and experienced in each setting, including observations, focus groups, interviews, free text from follow-up questionnaires, and a review of relevant documents. Observations included Circle sessions and traditional antenatal clinic appointments. Documents included maternity team meeting minutes, training workshop evaluations, field notes from facilitator reflection sessions, and facilitators’ written reflections. The focus groups and interviews were semi-structured and conducted with the midwives who facilitated the groups, the midwifery and other service managers, the women receiving group care, and those in the control group receiving standard care. Topic guides were used to provide a balance of openness and focus on the overall evaluation aims. Participants were encouraged to talk about their overall experience of care in a more narrative style, with some prompts relating to specific aspects such as postnatal contact with the group. We aimed to recruit participants from diverse groups in terms of ethnicity and socioeconomic position and include those with obstetric risk factors, since prior studies have shown particular benefits for people in racialised or socioeconomically disadvantaged groups (8) and also reflecting the findings of feasibility work that the women preferred the groups to be diverse (12). The participants could invite their partners to join interviews, observations, or focus groups if they wished. The researchers were from a range of backgrounds, including midwifery and anthropology, and were not involved in providing care, although some played a role in training provision and implementation support. Interviews were conducted either in person, via telephone, or via a video platform and were audio recorded and transcribed in full. Further details are provided in the trial protocol (16). Transcripts, observation notes, and open-text comments in survey forms were uploaded to NVivo 14 for analysis (https://lumivero.com/products/nvivo/).

Data were analysed thematically in two key steps. The first step was line-by-line inductive open coding. We then analysed the inductively coded data in relation to a “mechanisms of group care” framework developed by the study team in a realist review (18) (Table 1). This step was conducted iteratively rather than deductively to allow for new mechanisms and/or dissonant or disconfirming findings to be incorporated and the framework amended accordingly (20).

**Table 1:** Theorised mechanisms of effect in the literature [from Mehay et al. (18)].

Mechanism	Description
Social support	Bringing women together in a group and receiving continuity of peers provides the opportunity for building supportive relationships and social capital. Furthermore, trust can form to share experiences and disclose concerns, which can normalise pregnancy and encourage problem-solving, coping, and resilience, leading to reduced stress. This moves support to the community and reduces dependency on health services. Reference to social capital and community development.
Peer learning	Learning occurs through peers who are deemed to share similar characteristics as themselves (in some cases, sociodemographic, but more often the pregnancy experience). Information and messages from peers are seen as more salient, relevant, and personalised; therefore, women are more likely to act on that knowledge. Highlights the value of different sources of knowledge and expertise and that peers can be positive role models. This modelling leads to greater confidence in taking control of their own health by viewing others' behaviours. Reference to social cognitive theory and theories of behaviour change.
Active participation in health	Learning occurs through active participation in health and doing things for oneself, where self-checks, engaging in active discussions, and problem-solving place women at the centre of their own health. Shared health activities and engaging in women-led, group-based discussions supported more equal and trusting relationships between women and midwives.
Health education	A group setting allows more time for ANC education and for covering a broader range and depth of a health curriculum. Group ANC is theorised as a space to deliver behavioural strategies through specialised content (e.g., dental care, HIV support) and practical demonstrations to increase the transaction of “expert” knowledge and support for women to make appropriate choices for their health. Reference to behaviour change theories.
Satisfaction with care	A group setting enabled more time and continuity with a midwife and other healthcare professionals. Group ANC was seen as facilitating positive relationships between women and their healthcare provider, particularly where midwives are able to build relationships that are based on trust, leading to greater satisfaction with care, better management of risks, and increased engagement with health services generally. Furthermore, groups allow better joined-up care where other health professionals and invited speakers can attend groups to provide information (e.g., health visitors).
Health professional development and wellbeing	Midwives are able to provide richer and safer care with the increased time and continuity with the women, and by gaining the opportunity to develop their own knowledge with colleagues. This increases midwives' job satisfaction, which in turn translates to better care provided and reduced burnout.
Empowerment	Components such as interactive learning and peer group and relational continuity help support self-efficacy, confidence about health, seeking and using information, and decision-making. They may also help shift power balances and distance between professionals and clients, countering the hierarchy that is common in healthcare

## Findings

We first present a summary of the data drawn on for this analysis (Table 2), followed by a brief description of the case study sites. The thematic findings are then given in relation to the theorised mechanisms framework used in our analysis (Table 1).

**Table 2:** Summary of data sources for this analysis.

Type of data	CS1	CS2	CS3	Other: drawn from eight maternity services and external stakeholders	Total	Notes
Interview/focus group participant (intervention)	4	4	5	16 (of which 9 took part in a focus group)	29	*n* = 6 allocated to PC but left for a range of reasons. *n* = 8 high-risk obstetrically *n* = 19 social complexity
Partners (intervention)	0	0	0	4 (all took part in the focus group)	4	All partners took part in one postnatal focus group
Interviews with women in the control arm (standard care)	3	2	2	0	7	*n* = 4 high-risk obstetrically *n* = 5 social complexity (four had both social and clinical risks)
Interviews with midwives	5	3	5	10	23	All the interviewed midwives facilitated both PC and traditional care
Interviews with stakeholders	2	2	2	8	14	These included team leaders, community matrons, senior managers, consultant midwives, research midwives, and commissioners
Observations of Pregnancy Circles	2	2	8	2	14	
Observations of traditional visits	1	0	6	0	7	
Reflections by midwives	0	4	1	14	19	These include “reflection pages” from the PC Manual and field notes made by the research team during reflection sessions with the facilitating midwives
Free text from questionnaire at 35 weeks of pregnancy (FU1)	N/A	N/A	N/A	N/A	545	Out of 1,593 trial participants (34%)
Free text from follow-up questionnaire at 3 months postnatally (FU2)	N/A	N/A	N/A	N/A	475	Out of 1,593 trial participants (30%)

Illustrative quotes and excerpts are labelled as follows: CS1, 2, or 3 = case study site; Other = non-case study site; FGD = focus group discussion; feedback = feedback to service or in midwife reflection sessions; Survey = follow-up questionnaire in late pregnancy or postnatally.

In total, 24 (two-thirds) of the 36 women interviewed were identified as living with social complexity, of whom 6 had multiple disadvantages and 4 required an interpreter. One was under the age of 20, 16 were of ethnic minority heritage, and 8 lived in the lowest quintile of the index for multiple deprivation. Finally, 12 (one-third) had obstetric risks requiring additional scans and appointments, 10 of whom had both obstetric and social complexities.

## Implementation characteristics at each case study site

The case study sites were selected from among the trial sites for variation, including variations in local population characteristics and service organisation, as we wanted to explore how practice variations may influence the mechanisms of group care in different NHS settings. All the sites followed the key components of Pregnancy Circles (16) with variations in detail in response to their local contexts. Site 1 was in a coastal town with high rates of socioeconomic deprivation in a predominantly white community with high rates of teenage pregnancy and relatively large family sizes. It was described as having low levels of flux in midwives or women receiving care and reasonably high levels of antenatal midwifery continuity and family support. It had a single small obstetric unit and a small freestanding midwifery unit based in a rural cottage hospital. Site 2 was a suburban service with two obstetric units, each with an alongside midwifery unit (AMU), and a mix of more affluent and socioeconomically deprived neighbourhoods. It was characterised by strong leadership from consultant midwives during the implementation and long duration of the trial. Site 3 was a large inner-city service with two obstetric units and AMUs covering an area of high ethnic and socioeconomic diversity and a significant minority of women in maternity care with limited English proficiency (17%), with 33% born outside the United Kingdom (21). All three, based in South East England—reflecting the location of the majority of trial sites—were rated as “good” by the Care Quality Commission and had perinatal mortality rates within or lower than 10% of the national average.

## Mechanisms

The analysis confirmed that the theorised mechanisms we had identified in the literature were relevant and resonant with our data. Nonetheless, some adjustments were made during the analysis. First, continuity emerged as an important underpinning mechanism and time as an important underpinning component. Second, we combined the propositions related to learning under the overarching theme of a critical pedagogy, which is explained below.

## Social support and building community

Social support was integral to the design of group care and, as indicated under peer and interactive learning and continuity, the approach to information provision and the consistency of participants and facilitators were contributors to this. Many participants spoke about this in interviews or added comments in their follow-up questionnaires; for example:

“This is my second pregnancy and i [sic] feel the antenatal care support i [sic] have received as part of the pregnancy circles is far superior to that of the 1:1 sessions I had previously. It is great to be a part of a group of other women and I enjoy the fact that we receive the usual 1:1 midwife care but also discuss other topics as a group.” (Survey G52, intervention FU1).

“This was fantastic I got to meet new mums and able to ask lots of questions and feel ready to be a mum.” (Survey C06, intervention FU1).

This was consistent for women who did not see themselves as extroverted or confident:

“I would totally recommend the pregnancy circle option to anyone especially if you are quite shy and anxious like me it's a good way to not feel so alone during pregnancy and then have friends to do things with after babies are born.” (Survey H25, intervention FU1).

Midwives commented positively on the ways they felt the women in the groups supported each other and their observations highlighted a range of supportive interactions, such as helping each other with self-checks, offering refreshments, and providing words of comfort or validation when participants had worries or concerns. As one midwife explained:

“… one of my ladies, she had some mental problems, of course she's struggling more … so when we were meeting up in the group, she seems like coping a little bit better, because she can communicate, she can talk, she didn't have the very big family and her partner is at work most of the time.” (Other, interview with midwife 3).

The women were also observed to play facilitating roles in some cases, such as drawing their peers into discussions or those with more experience providing insights from experience and reassurance to first-time parents:

“One woman does a small demonstration of cloth nappies for the others in the circle- she has brought with her cloth nappies, cloth liners and explains how she plans to use them, how to wash them, how many you need to buy, cost and how to make this cost affordable (buy second hand). Another has brought printed handouts on cloth nappies which has details of price, availability, brands. This leads into a discussion about individual choice, environmental issues, cost. One woman shares that cloth nappies used to be very common in Africa but more recently, there has been a rise in disposable nappy use.” (CS3, observation 1).

The potential to mitigate birth trauma was also mentioned by the midwives and women. The women talked about how feeling better informed helped them to cope with difficult births and interventions; for example:

“I would recommend it to everyone. It was absolutely fantastic. I was terrified of having a c section [sic] but pregnancy circles helped me feel a lot better about the procedure. I ended up being induced and had an emergency section due to the cord compressing on my babies [sic] neck. I was still nervous but felt a lot better after our talks in pregnancy circles.” (Survey P46, intervention FU2).

The midwives also highlighted the value of the postnatal session to follow up with the women and link them with health visitors or other support services, in addition to the peer support from the group. One midwife flagged the loss of social support when a group was discontinued because of the COVID-19 pandemic, but noted that the women continued to stay in touch via a WhatsApp group.

The participants themselves described hearing from others and doing things together as helpful in encouraging healthy behaviours, for example:

“We all went with yoga balls, and we talked about exercises and just when I guess when you hear that all the people with you are going to the same thing, practicing these exercises or going through these sessions, it makes you a bit more [sic]. At least I felt like I could probably do it too, like, so I signed up for yoga sessions and ended up walking more because people are going for walks with the even with their newborn. From [sic] my culture, people don't really get out with their newborn to like 2–3 months, but the people in the Circles who were going for walks like in a week's time I'm and I think that's really good.” (Other, interview with woman 16).

There was also evidence of community building through continuing the support and connections established during the Circles:

“It's really nice how everybody is still very much in touch and there are plans every month and if there's something that somebody's worried about or ‘is this normal’ for because I think all in our group, everybody's a first-time mum. So everybody's a bit like ‘ohh is, is this expected? Is this normal?’ Things are changing every day and it's, it's nice that the [WhatsApp group was] proposed and even and everyone's open about their experiences.” (Other, interview with woman 1).

In most cases, the group was experienced as a safe space to share worries and gain support and information:

“This is my second baby and I feel that I have had more chances for open and honest discussion and have been given a lot more advice.” (Survey H02, intervention FU1).

Social support was not dependent on homogeneity of personal backgrounds and experiences so much as the shared pregnancy journey. Diversity in the groups was generally viewed positively by participants and midwives; for example, these midwives discussed their observations on diversity:

P1: Because everyone's bringing their different experiences, aren't they … Umm, it was … and I think they were really tolerant of each other, as well, because they were very different, weren't they?

P2: Mmm

I: In what ways were they different?

P2: Umm, I would say one lady had a, maybe a bit more of a socially-deprived background.

P1: Yeah, which she spoke very openly about, didn't she?

P2: Yup, yup … Umm…

P1: One lady a bit more middle class…

P2: Yep…

P1:  … with, you know …  Then there was a younger girl, first baby

P2:  … and then … yeah, one girl, bit younger, having her first baby …  and I did wonder at the beginning how that would impact on her, having everyone else already had a baby, but.

P1: She was actually the most vocal out of the group, on the WhatsApp group, isn't she?

P2: She is, yeah, yeah … I think she's got a lot from the other mums. (CS1, Joint interview with midwives 1 and 2).

Groups with a good level of ethnic diversity were observed as enabling women to share and compare cultural knowledge and practices, including healthy food and weaning, with midwife facilitation and this was echoed in women's responses. For many racialised or otherwise marginalised women, Circles was a place of cultural safety, in sharp contrast to descriptions of other hospital services, which can be experienced as inaccessible and stigmatising:

“No one is there to listen to you. When you call, they say sorry can you ring this number. When you call your GP they say sorry we can't tell you anything or how to get through to your midwife, sorry ring this number and when you ring that number they say you have not been assigned to any midwife, it makes you feel tired, it's so heart-breaking, so that group with my first pregnancy was fantastic.” [Other, interview with woman 14 (Black African)].

“I definitely don't feel like in my labour they listened [to me], but I also think I wasn't in a position to talk for myself at times … I don't know if my experience would have been different if I was a white person maybe … I don't know if it's health, I just don't know if it's because we're not as prepared, I don't know if it's because we don't always get the best treatment.” [CS3, woman 2 (Black African background receiving standard care)].

Nonetheless, a small number of participants highlighted difficulties with feeling that they fitted in with the group. For example, some women with a high BMI, those from a minority ethnic background, or those of an older age felt different from the other participants if the group was not diverse overall, and some expressed that they were hesitant to raise questions around issues such as weight management:

“Know what I mean? Like, my voice is never going to be heard, as the Black woman who's overweight, having my third baby, in a room of white women that are not overweight and having their first child.” (Other, interview with woman 6).

A lack of skills and confidence to facilitate a discussion of sensitive topics in a group setting was observed in a few Circles, indicating a need for further development support among some midwives:

“The midwives also discuss that they think it's inappropriate that a woman with such a high BMI (over 40) is in the Pregnancy Circle—the language used “she knows she shouldn't be in here” (emphasis is the midwife's). They talk about how they find discussing diet awkward with her when the other women in the group are visibly not obese. … One midwife is visibly blushing and is very uncomfortable discussing this. The midwives also share that they think Pregnancy Circles might not be a suitable place for high-risk women because things take longer and often require further referrals that take more time.” (CS3, observation 1).

Although there is evidence that in standard individual care, midwives lack skills and confidence in addressing potentially stigmatising topics sensitively (22, 23), this highlights that additional skills may be needed to facilitate psychologically safe group discussions and highlights the importance of midwives participating in training workshops and follow-up reflection sessions.

The midwives felt the group model could be particularly helpful for women who are socially isolated, for example:

“She doesn't have many friends, as well, so she is learning about pregnancy and about, umm, we had a woman who was breastfeeding. She came and breastfed her baby and talked about infant feeding in the group, and I think a lot of women hadn't seen that before… So, for the vulnerable and the people who haven't got mothers around them, or role models, or, and we've got two multips and four primips, so they're sharing together, their experiences.” (Other, interview with midwife 2).

Participants commented on the value of this support in the early postnatal days and for the care of the baby. The following woman, for example, talked about support from the group via WhatsApp when her baby was suffering from constant colic and crying:

“I probably would have rushed to hospital because I didn't know what to do, but because something so simple and somebody else was going through it and it [giving some drops] didn't seem like a, a very invasive anyway.” (Other, interview with woman 1).

The contribution to postnatal and social support was also observed from midwives and participants; for example:

“Midwife uses this [button support activity] as an opportunity to discuss health visitors and community midwife schedule of postnatal visits. Women share networks of support locally with other women: baby groups, Children's Centres, baby and toddler activities.” (CS3, observation 1).

Approaches to group care have varied internationally regarding the level of involvement of fathers/birth partners. In the Pregnancy Circles approach, each group was encouraged to discuss the level and timing of partner involvement during the first session. The groups varied, therefore, with some feeling they needed time to bond as a group first and then include partners in the later sessions. Others allowed limited or more active involvement and a few chose not to involve partners at all, which could be disappointing for some of the participants involved:

“I've had a really positive experience but have missed having my partner more involved. And he has commented on this also.” (Survey P603, intervention FU1).

The women sometimes preferred for their own partner to be included, but this was not the case for others. Some groups had higher levels of partner involvement and observations suggested this could work well with open discussion, with the opportunity for participants to get to know each other and feel safe to discuss sensitive topics in the group space*.* However, we do not have sufficient data to investigate how the mechanisms varied in groups with differing levels of partner involvement.

## A critical pedagogy

The mechanisms related to information and learning identified in our realist review, namely, *peer learning, active participation in health,* and *health education,* were closely interlinked in our study. We combined these under an overarching theme of *critical pedagogy*, which is a philosophy of education that aims to address social inequalities through critical thinking and social action. This concept draws on the theories of Freire (24) and hooks (25), who argued that to be effective (deep rather than surface learning, which implies deeper understandings and greater retention of knowledge) and transformative, pedagogy must be participatory, involving people actively, and recognise that all have contributions to bring to a learning process with strengths and needs or vulnerabilities. Although the concept of health education draws on more behavioural and transactional approaches, which differ from the philosophy of more active and interactive approaches that we align with in critical pedagogy, the analysis highlighted ways in which the participants felt they had acquired health knowledge and support for healthy behaviours, which supported their confidence in being able to care for their health.

The training workshops provided to the midwives who facilitated the Pregnancy Circles in the trial were designed to support midwives who have been schooled and socialised in a more didactic lecture-style approach to develop their skills in critical pedagogy, through role modelling a facilitative and interactive approach to information provision, including active learning techniques such as role play, reflection, interactive games, and active discussions, rather than a lecture-presentation style. Although not part of our qualitative data collection, we noted that in the workshop evaluation forms, the midwives commented on how this approach helped them develop skills in facilitation but also challenged their traditional ways of thinking, enabling the philosophy to “click” as something they could put into practice. One stakeholder commented on the 'shift in thinking' involved:

“That's probably the shift in thinking that needs to take place, because at the moment I think those that haven't done Pregnancy Circles probably look on it as some sort of antenatal education type offering, and it's very different and that's what we probably, that sort of culture change is something that's needed.” (Other, interview with stakeholder 7).

The midwives suggested that this approach could more effectively promote health by improving the participants’ capacity to process, understand, and retain information; for example:

“… a longer lasting health promotion benefit. And actually, you know, perhaps a longer support outside the pregnancy group which is enabling these women to look at what is the data, what are we preaching about, and actually taking that story on board, a message on board, a bit further down the line … their mental health has to be improved as well, because often that can be quite daunting, hearing ‘well how’s it gonna affect my baby if I've just been diagnosed with gestational diabetes?’, and it, we don't have enough time to devote to that patient and their expectations often aren't met, so they, you know, we usher them in, usher them out, give them a message, and that's it, job done.” (Other, interview with midwife 9).

Another midwife contrasted the level of active participation and discussion in the Circles with the usual parent/antenatal education classes:

“… we have ten women and partners, and we do three sessions and they all just literally sit there. And you ask them to kind of go around and say something about themselves, and they just say what the person before them has said, kind of thing, and it's really difficult ….” (CS3, interview with midwife 1).

Group care sessions (as highlighted in the name, Pregnancy Circles) used a circular room layout for the discussions, typically with a table to one side where the women (and sometimes birth partners) in the group collaborated in taking and recording their own routine measurements, such as blood pressure, urine testing, and, latterly, carbon monoxide monitoring. Individual clinical checks and brief discussions were usually conducted on a mat in a quiet corner, while the conversation continued in the circle. The experience and impact of this collaborative approach were highlighted in a number of interviews with female participants, for example:

“Every time we come, the first thing we do is how to do a urine sample and dip it and check and if you still have problems reading it, they tell you this one means this, this one means that they showed us where we can record it, but even when you are confused, you also call them they are there. It makes you feel belonged [sic], it makes you feel involved in your pregnancy journey, it makes you feel I've learned this, I have learnt that.” (Other, interview with woman 14).

The midwives also commented on the self-checks as being valuable for learning, and the surprise of other professionals that women could be this involved. This senior midwife commented on how, when the women who had been in Pregnancy Circles attended individual visits, they still expected to be more active in their own care:

“They're going, oh, where's the dynamap? [sic; blood pressure monitor] Oh, OK. It's over there. OK, off; they do their blood pressure. And then … they're like, right, so, where am I going? You know, you're like, oh, that's a good point. I need to take you to the sluice so that you can tip your urine and do it yourself. And obviously, the clinic sisters are like, they're doing what? They're going round? … So yeah, that was brilliant because they've got these enabled skills already. And they were like, yes, I know what I'm on about. I'm doing this myself.” (CS2, interview with stakeholder 2).

Peer learning was achieved through guided activities, by supporting each other during self-checking activities, and through a facilitative approach to information-sharing, including techniques such as reflecting questions back to the group rather than simply answering them directly and actively encouraging participation in discussion on pregnancy, birth, adapting to parenthood, wellbeing, and related topics. This approach aims to tease out a participant's existing knowledge and ideas (including information sources) for more exploration and group discussion, and to support peer interaction and learning. The women often commented on the knowledge gained from being together and the reassurance this provided; for example, a woman with limited English proficiency said:

“And yeah, we would talk about body parts and things like that which would be happening to us at the moment, ‘cause like a lot changes in your body when you're growing a baby and it was nice to know that’ kay, it's not just me, it happens to nearly every woman that's going through pregnancy.” (CS2, interview with woman 4).

The responses also illuminated how this style of learning, in a context of high socioeconomic disadvantage, could support self-efficacy and health knowledge, as illustrated in this exchange:

I: Did you find … because you've had many babies, did you find that you had a lot to share with the others?

P: Yeah, yes, I could share a bit more of my experience and yeah…

I: Mmhmm, was that…

P: … all the horrible bits! But there are good bits as well (laughter)

(CS1, interview with woman 6).

The groups were designed to be inclusive of those having first or subsequent babies, those with different levels of risk, those from diverse socioeconomic or ethnic backgrounds, and those of different ages. How far this was achieved in practice varied on a local basis, but a principle of peer learning was that the women involved would have a range of experiences and knowledge to share and would ask questions others had not thought to ask, with skilled facilitation by a midwife to support effective information provision. Participants responded positively to this diversity; for example:

“I thought it was really good, really good. I really enjoyed it, ‘cause what kind of helped me, obviously I know that each person's delivery and labour and all sorts is like, pregnancy alone is all different. But all the other ladies were already mums, I was the only one in there that was first-time.” (CS1, interview with woman 7).

The midwives also commented on how the women with more risk factors still wanted to participate in most cases, despite having a number of additional appointments. For example, a senior midwife commented:

“She'd had her scan for twins when she still said, yeah, I'm still coming … . knowing she was going to probably end up with the caesarean section, but she thought it would be a good way to share all that sort of stuff. But it was quite nice. Because then when you're talking about your first baby, first baby; so she's like, oh, I had a water bath … So, but that's their experience is telling the women that, that's not me.” (CS2, interview with stakeholder 2).

The women were observed discussing a wide range of pregnancy, birth, and postnatal issues, with varying levels of introduction or input from the midwives, including topics as diverse as healthy eating, maternity rights and benefits, what equipment to buy, staying at home in early labour, epidurals, physical recovery, feelings, and adapting to parenting postnatally.

The observations illustrated how sharing health information, including the advice received from friends and family, could help participants consider information from different perspectives and weigh up the information they were exposed to. In one group, for example, the midwives were observed discussing “cot death” (sudden infant death) and care and sleeping arrangements, advising no swaddling, bed bumpers, or pillows:

“Women are surprised about swaddling and discuss conflicting information from friends/family/other healthcare professionals.” (CS3, observation 3).

Nonetheless, we observed that a shift from a didactic lecture style to a critical pedagogy approach presented challenges for the midwives, who needed time, support, and motivation to develop their skills and confidence using a more facilitative approach. Most were new to this way of working and some were anxious or reticent. Some struggled to develop an approach of “reflecting back” questions to prompt more interactive discussions; instead, they provided direct answers, highlighting that even with volunteers for a new model, time and support are needed to establish a different way of working. In some cases, particularly where there was a lack of continuity or a long delay between the training workshop and starting circles, midwives were observed to move away from a “circle” approach to one more closely resembling a more linear type of classroom layout, such as standing up while facing the participants, or both midwives conducting clinical checks while the women waited, rather than combining a facilitated discussion and individual checks throughout the sessions; for example:

“Both midwives are still out of the circle writing notes and taking bloods, so the women are leading on perineal care, including massage and pelvic floor care. One multip shares her experience of episiotomy and what she used to help it heal afterwards. Primips in the group ask what an episiotomy is and why it would be done, the woman is not clear about why she had an episiotomy. Midwives do not contribute to this discussion.” (CS3, observation 1).

“MW2 speaks openly to MW1 about the women being out of the circle too long and expresses her frustration with MW1—says she has mentioned this to other midwives she does other circles with- there's no need to have women watching you write up notes, they can be back in the circle participating whilst you write up and this is not how they’ve been trained to run the circles and they are always going to overrun if the future sessions are run like this. MW1 shrugs her shoulders slowly and says slightly awkwardly to me that she is a 1–2–1 midwife, and that women need the private time.” (CS3, observation 8).

Even in such instances, however, interactive discussions and peer support continued:

“Women lead other [sic] discussion on pumping: how do you feed twins? What if you spend the day out? Women ask about feeding cues, on-demand feeding vs formula feeding … Women start sharing recipes and diet advice whilst the midwives are preoccupied with bloods and notes. One woman (high BMI) has her BP rechecked by one of the midwives with a manual cuff. Women give encouragement to her about stopping drinking coke [sic] in this pregnancy.” (CS3, observation 1).

One midwife described using one woman's diagnosis with gestational diabetes as a positive opportunity to share knowledge about managing health conditions and diet in an inclusive way:

“… we discussed it in the group—what is gestational diabetes, how do you detect it, who is at higher risk, and things like that.” (CS1, interview with midwife 5).

This contrasts with the case described above of a midwife lacking the skills to facilitate a supportive and informative discussion about a topic such as weight management.

## Engagement and satisfaction with care

While a range of external factors could affect care attendance, and for some, the longer session time in Circles presented a practical barrier, almost all the women talked about their care experience positively, saying that they felt more involved, had a higher level of support and information access, and felt they would opt for this form of care again or recommend it to others.

A number had fewer Circles than expected because of the COVID-19 pandemic lockdowns, but still valued the limited experience:

I: So if you were to have another baby, and if you were to get the option of Pregnancy Circles or traditional care, what do you think you would choose?

P: Pregnancy Circles, I definitely would, because although I didn't have it for long it was very useful … . In terms of, like, the information and feeling that you've come away knowing more for yourself rather than just relying on the hospital to tell you certain things, you know, you get a well-rounded knowledge. (CS3, interview with woman 6).

In a smaller number of cases, including some who had additional medical visits or complexity, it was more difficult to maintain participation; thus, their engagement with the group was reduced. However, they valued the social support and interactivity of the group and the chance to receive “normal” care and were motivated to attend.

Those who participated had agreed to group care within the trial, with the possibility of randomisation to group or individual care. We were not able to interview those who declined, but the recruiting midwives recorded the main reasons for declining, which were usually practical, such as difficulty in arranging childcare or leave from work for the duration of the sessions (26). Interviews conducted with the Circles participants who withdrew from the study also confirmed that this was usually for practical reasons such as childcare problems or difficulty with session timings. However, one woman found the lack of privacy in the one-to-one clinical checks difficult and another left because the midwives could not obtain an interpreter for the group.

Therefore, positive or at least neutral expectations of group care should be anticipated. One woman reflected on her expectations being met, saying the following:

“I just thought it was a really good idea, I think it's nice that you've got all these pregnant people together and we could all discuss our … and I found out so much that I didn't know, I've had … this is my seventh, and I didn't know half of it …. ” (CS1, interview with woman 6).

She went on to mention that she did not know the signs of pre-eclampsia or gestational diabetes or the purpose of checking blood pressure prior to taking part in Circles, despite six previous pregnancies in traditional NHS care.

The women in the control group, in contrast, often indicated more basic levels of satisfaction with care, as the following questionnaire comment illustrates:

“It was just a form of routine check for me and the baby to ensure everything was okay.” (Survey K74, control FU1).

This could be couched in allowances for the busyness of the midwives and demands on the NHS service; for example:

“I just got more information only if I am asking further questions otherwise we are going through general basics [sic] checks. But midwives have been helpful and very nice so nothing to complaint [sic] about it. Just I saw a different one anytime [sic] I went so no one remembered or even know [sic] me. For NHS antenatal care services I think this is good for at least someone like me with no complications during my pregnancy.” (Survey P606, control FU1).

“When I had questions, the midwife would answer them, though it always feels rushed.” (Survey R119, control FU1).

Such comments illustrate that women may often limit their expectations of NHS care in a context of constrained resources.

## Health professionals’ development and wellbeing

This mechanism was less well-developed or explored in the wider literature that informed our analytical framework, but emerged as an important theme in this study.

Group facilitation using an interactive approach was very new for all the midwives involved, and, as noted above, a key challenge was facilitating information-sharing among the women without becoming directive or correcting them, while still ensuring accurate information was transmitted. Exploring sources of knowledge and understanding is complex and the wider literature suggests the skill is underdeveloped among many health professionals.

Some midwives had initial concerns about the ability to provide accurate health information using this approach; however, they were generally reassured by their experience:

“When you let them talk, people will say something and then, you know they've said something that's not quite right, but you let the other members of the team, or the group, sorry, discuss whether or not they agree with what they've said, or whether they, that sort of thing, and then they kinda come up with their own conclusions themselves … So I feel like a lot of women are learning a lot of things that, from each other, not just from us, which is great.” (Other, interview with midwife 1).

Midwives talked about the positive rewards of feeling they were providing good care, seeing the social support, and having relational continuity, suggesting in some cases that this was returning to what they felt was proper midwifery:

“Pretty much all of us have said that we want to continue this because we think it's a brilliant way to deliver antenatal care. I can't, sometimes I can't quite believe how much I've covered in one hour, with nine people, they're getting a substantial amount of information, and they're building really good friendships with each other.” (Other, interview with midwife 1).

This was also observed by senior midwives:

“A lot of midwives loved it because they thought they were, umm, they told me they felt like they were doing proper midwifery, they were doing the whole shebang. It wasn't like ‘oh, here comes another 14-weeker’ … the exact same thing, rota, repeat, repeat, repeat.” (CS1, interview with stakeholder 1).

The midwives talked less directly about their own enjoyment of working this way once they had gained skills and confidence, so this was largely apparent indirectly through observations and enthusiasm to continue. Nonetheless, some commented on how much they anticipated the Circles:

“I actually really like working in the model, the pregnancy care, umm, Pregnancy Circle model, and with the other midwives. I've learnt loads, and I actually think it's much a nicer way to work.” (CS3, interview with midwife 1).

“… even at home, I was like ‘oh I've got a brand new Pregnancy Circle starting today’, and it, I was excited about it and then to come in and go ‘well actually no, it’s cancelled, you're doing bookings all day’, ‘oh great’.” (CS3, interview with midwife 3).

Midwives typically provide individual care and work alone in busy services, with little time allocated for peer discussion and review beyond specific cases and workload planning. Group care involved working together, an unfamiliar experience for most, which some reported feeling nervous about. Several midwives spoke about the benefits of this approach in terms of sharing knowledge, using complementary skills and strengths, and supporting each other, in a way that paralleled the interactive learning principles of the group for the participants; for example:

“Before we started, when we had just the prospect of doing Pregnancy Circles, I felt a little bit on edge and I felt a bit apprehensive, and I thought ‘ooh, is this just another thing that I'm going to have to try and work into my diary? How am I gonna manage my time?’ Umm, but certainly once I've started, and after doing a couple of circles, that anxiety certainly goes away, and any worries you are having, I feel like they're shared between the two of you … umm, or if you've got a lady, a patient, who perhaps needs a little bit of extra care, for whatever reason, uhh, you can share that between you rather than having that all upon yourself.” (Other, interview with midwife 4).

Others described benefits in developing their working relationships, their learning, and finding they had complementary skills:

“I was quite daunted by that [working together] I felt like, a bit like ‘oh my goodness, am I saying the right thing?’, and then you kind of realise that everyone feels like that, and you learn things from what other people are saying, and they also learn things from what you're saying, and there's definitely things that, you know, different midwives are better at.” (CS3, interview with midwife 1).

One stakeholder also suggested the approach could help break down isolated ways of working:

“I think they [the midwives] really like knowing what other people are doing and how they might be doing things differently that might be having a positive effect in another way, as well as sharing their own experiences. So I think that was also important, you know, in terms of the working in silos that's so often so common in maternity services in the UK, just very much opens everyone up.” (Other, interview with stakeholder 7).

Some midwives reflected on the impact on their own skills and knowledge; for example:

“I think continuity has definitely changed my practice as a midwife, but Circles as well because I think the main, the main thing and the way that I can sum it up is just the role of the midwife and what the perception of that role is versus maybe what it's like in reality. … it's like right, these are the checks you do at these appointments and this is the information that you give at those appointments and it's not tailored at all to what those individual people need. Whereas when you're kind of giving them the autonomy over the clinical checks, that's one thing it takes away from me … but you know, if I can be instrumental in helping them in their decision-making processes and their birth preferences and, you know, bring in their child into the world, for me, that's so much more rewarding, that's what midwifery is about.” (Other, interview with midwife 10).

## Empowerment

The view that this form of care is empowering was cited in the wider literature review as an overarching mechanism by which group care may achieve wellbeing benefits. However, the details of this were often underexamined (18). Although empowerment can also be considered an outcome, we considered it to also function as a mechanism through which more specific public health outcomes may be enhanced. Our analysis elucidated the ways in which the model supported empowerment via mechanisms such as social support and critical pedagogy. Empowerment was referred to directly and indirectly by Circle participants and midwives:

“I knew about different places because of my work, but Circles empowered me to actually go to them, that its OK to ask for help.” (Other, interview with woman 8).

“I feel like I had more knowledge now going into it, so I knew what I wanted to do when I went in and understood why I wanted to do it.” (Other, interview with woman 13).

“I think it's because it was in a group, it's being able, I'm not really one to, like, jump up and ask questions or query anything. But because they were all doing it. I was like, Oh I can join in now.” (Other, interview with woman 2).

This was also expressed by the midwives and service leads; for example:

“It made me feel like ‘this is your time, your important time’, so I wasn’t the person who had all the answers. In fact, often the case, you know, something would come up and it would be a shared experience of someone in the group. It wasn’t necessarily me giving all the answers, it was, you know, I was empowering them to sort of be resourceful with what they could come up with… I can’t tell you what we’d achieved, but it felt like we’d achieved something with that group.” (Other, interview with midwife 9).

“I felt it broke down a lot of barriers, between the midwives and the women, it was quite, not, even though it was a professional interaction and clinical aspects were taken into consideration, the fact that it wasn't like timing within a certain frame, it wasn't rushed through, I felt for me the midwives felt it was time well spent.” (Other, interview with stakeholder 1).

“As a team they're getting a reputation for developing ‘strong willed’ women.” (CS2, reflection session 2).

Some also spoke about empowerment for themselves as midwives, even though adopting a more facilitative role could be assumed to mean a loss of power:

“I really enjoyed doing it. I felt it empowered me as a midwife. I felt I learnt a lot about me, and I enjoyed every bit of it really, enjoyed working with my colleague.” (Other, interview with midwife 9).

The Pregnancy Circles approach, except in one NHS service, did not extend continuity into intrapartum care. As this was a new approach that was being trialled in each service on a limited scale, many professionals who were not directly involved were unfamiliar with the model and had not participated in the facilitation workshops or planning meetings. Thus, the women may have encountered dissonant approaches to informed choice and support during their labour experience. One woman, for example, was observed in a postnatal session describing the mismatch she experienced when in labour:

“W4 said that the midwife at her birth was “not a good personality fit for me. I didn’t feel listened to”. The midwife advised her to have the augmentation drip, but she wanted to avoid epidural because of a spinal problem so she “sent the midwife out of the room, spoke to my partner and made a plan”—pethidine and wait & see, which worked. ‘I know there were alternatives due to the discussions we have had and my own reading’.” (Other, observation 2).

While this excerpt illustrates that the woman felt empowered enough to assert her wishes, this did not apply to all those who encountered a different approach in other aspects of their care. Another woman, for example, said:

“I did not have a good experience of birth, felt highly pressured into decisions I did not feel comfortable with and I was later told I could have been put on a different pathway that would have given me more choice or avoided me having conversations with staff who gave me misleading facts at the hospital. I did not have a clear picture of options, as what I believed I could do was different when I got to hospital.” (Survey J03, intervention FU2).

This suggests that the impact of empowerment on birth experiences or outcomes may be more limited without continuity across the whole care journey and consistency of philosophy and approach across service providers. Nonetheless, our qualitative findings on empowerment were concordant with the analysis of the trial outcomes as we found that the participants in Pregnancy Circles were significantly more likely to feel that they were always involved in decisions about their care, that they were well prepared for labour and birth, that they managed very well during labour, and that they were confident in caring for their baby in the first week after birth (26).

## Relational continuity

To support the principle of peer and interactive learning and for the group to function as a safe space where experiences or worries could be shared, continuity of facilitators and of participants emerged as an important underpinning component. Each service involved in the trial identified specific midwifery teams (usually but not always community midwifery teams) that would provide group care, and rotas within the teams were planned so that the same two midwives would normally facilitate care for a specific group, with a third midwife identified as back-up in the event of holidays or sickness. Assistance was provided by the research team to schedule this new way of organising antenatal care. One service opted for its existing midwifery continuity of carer teams (a caseloading model providing continuity through antenatal, intrapartum, and postnatal care) to provide the group care, thus piloting how to combine these two models within the setting of the trial. In some settings, a midwifery student, maternity support worker, health visitor, interpreter, or bilingual health advocate was included, also with continuity.

The observations of the groups and interviews with the facilitators highlighted several features of continuity. The midwives commented on the opportunity to get to know and understand the women in their care more deeply. This also applied to the midwives working in the established continuity teams, some of whom described getting to know the women even better through observing their interactions within the group. The midwives felt that the women in the groups were able to develop bonds and feelings of safety that enabled them to participate more actively and to disclose worries, concerns, or details of personal situations; for example:

“We were talking about emotional well-being and one of the girls in the circle was very much, oh, you know, I had it [baby blues] before. I, I don't know how I'm gonna cope. And she'd started crying. Anyway, so [the Health Visitor] shared that piece of information with her and said, you know, we're here to help. … So obviously all the other girls like bounced on her to say, oh, no, no, you're, you know, all together they all came up with these different ideas and suggestions they were going to go off and do group swimming classes together and all that.” (CS2, interview with stakeholder 2).

Another commented on the feelings of safety that developed within the groups:

“I think it also helped their mental health ‘cause it allowed them to really have dark, deep conversations about how they were feeling, and what they each recommended that helped them in terms of, you know, the morning sickness, or feeling tired, or work pressure; it allowed them to sort of share those personal stories at a deeper level and have that shared wisdom of conversing with each other in a safe room.” (Other, interview with midwife 9).

In interviews, the participants highlighted the value of continuity, both regarding the midwives and their peers, echoing the midwives' observations that continuity enabled a feeling of “safety.” Furthermore, they highlighted the bonds within the groups that, in turn, supported other mechanisms such as peer learning, social support and community building. One woman who was receiving individual care commented on how one may be more able to share feelings in a group:

I: What do you think about being in a group of other women who are going to have a baby at the same time as you? Do you think that might have helped you?

P: Yeah, yeah. Because maybe you're not going to be shamed to talk about your feelings, what do you think … You can talk to the woman, and she can share her experience, and I can share my experience all the way … you learn. (CS2, interview with woman 3).

The participants welcomed the chance to receive social support from the others in the group through sharing knowledge and experiences, with one woman with limited English proficiency saying:

“You know, working in the group of women all in the same condition was very helpful because we used to talk to each other and any problems we were experiencing individual, and whether any itching, any health problems, anything like that.” (CS3, interview with woman 4).

We did not identify any data in the women's interviews to indicate any negative aspects related to continuity, either of facilitators or group participants. However, the planned level of continuity of facilitators was not always maintained and, in some groups, a smaller-than-planned group size or participants with higher levels of social and/or medical complexity disrupted the level of peer continuity. Even in such cases, we found that the participants often maintained continuity of peer support via WhatsApp. Some women with higher medical risk factors had numerous additional appointments, which disrupted their capacity to attend the group sessions. This was also influenced by mixed messages from other maternity professionals who were not always aware that group care can include women with varying levels of risk and advised that they should no longer participate:

“Well [it was] very much like ‘okay, from now on you’re going to be coming to this clinic every 2 weeks, you can no longer go to the Circles’. Erm, ‘cancel- if you've got Circle appointments on your app, ignore them, just come to these appointments’” … But I was quite keen to get back to the girls and like, let them know what was going on. Erm, and then eventually they were like, ‘oh yes, yes you can still go to the Circles’, so I continued going to the Circles. In general—this is nothing to do with the Circle—I think the only consistent people that I saw throughout my pregnancy was the Circle. Like every time I went into the hospital for something that I was seeing somebody else. I don't think I saw anybody twice … Whereas when I went to the Circle it was nice that they would follow up ‘okay you said that this happened’, or ‘what's going on with that’.” (Other, interview with woman 6).

The midwives also valued the continuity and co-working in terms of information and care planning; for example:

“We'd always have a cup of tea, sit at the table and just get into the zone ‘this is our Pregnancy Circles’, chat about who we were gonna see, what their blood results were. … so we could have that all sorted and planned in our head so that we could just let the group run and discuss privately the results and bits and bobs.” (Other, interview with midwife 9).

## Time

Time emerged as an important underpinning component, which, along with continuity, was consistent in the data as an enabler of the mechanisms, confirming one of the key elements of the Pregnancy Circles core values and components model (16, 18, 26). The sessions were 2 h long compared with the typical 20 min for individual antenatal visits, enabling more extended discussions. This is connected to the benefit of continuity, as the participants were able to return to and develop discussions and understandings over time: thus, this dimension of time emerged as an important aspect of how continuity, rather than fragmentation of care, functions. One midwife, for example, reflected on how being able to discuss health issues within the group and also return to them over the course of care could help participants to “digest” health messages:

“There's a bit more of a valued conversation because we had more time to devote. And that felt, you know, also it felt that we weren't the ones giving the message. The group shared it and the group were able to review and reflect what worked for them, so it wasn't, we weren't just being strictly dictatorial, the group was able to digest the information and work out how they could trial different things, whether it was just having a daily walk or going for a swim. And in fact, a couple of them did meet up for walks and swims and yoga classes, so that worked, yeah.” (Other, interview with midwife 9).

Another, who had expressed some concerns initially about managing group dynamics, also commented on the importance of time and continuity:

“The advantage to the Circles is you do have more time to discuss things, and that is something that has been really good, as a midwife, is that I do feel like I've got to know those women better.” (CS3, interview with midwife 1).

Time constraints in traditional care were similarly a common theme in our data, and were perceived as a root cause of sub-optimal care by both the midwives and the women. Even the midwives with Pregnancy Circles experience who wanted to transfer those skills to their traditional clinics found that they were constrained by short appointments. The midwives talked about the increasing range of areas and specialisms they were expected to discuss in a short time within individual visits:

“You've got all these different people that see antenatal care priorities quite differently, and when you're doing one-to-one or traditional care, it's really hard to convey all of that information in a really nice way to the women, kind of, without just giving them bullet points of information; whereas in a circle, you know, you can do a whole session on whatever might be particularly important to those women at that time, and … so I feel like it's much easier to have all those conversations that other people want you to have, to deliver all the information and still get the clinical care done.” (Other, interview with midwife 4).

## Discussion

While previous studies have focused on satisfaction with and experience of group care (6) and on attendance or clinical outcomes, and others have argued that group care will increase community building and empowerment (18), few studies have used a realist-informed approach to explore the mechanisms by which providing care in this way may lead to more positive care outcomes and experiences. In this analysis, we were able to identify such mechanisms in the context of universal NHS care from the perspectives of care providers and participants and the key underpinning features of relational continuity and time. Empowerment was confirmed as an overarching mechanism that linked the elements of social support and a different approach to learning, which we have characterised as critical pedagogy, with a more active and positive experience of care that enhanced the participants’ sense of confidence and feeling well-informed. These qualitative and conceptual findings were concordant with the preliminary quantitative findings of the Pregnancy Circles trial (26). Our analysis identified that empowerment was also relevant for the midwives who facilitated the Circles, suggesting that this was mutually constitutive rather than the empowerment of one group implying a loss of power for another. Nonetheless, the findings elucidate differences in this process for providers and participants, as the key elements for the midwives included co-working, learning feedback through continuity, getting to know the participants, and understanding the influences on the participants' health. Although a more interactive and participant-shaped approach may appear to reduce professional control, the midwives in this study spoke of feeling greater autonomy and scope in their practice.

**Figure 2:**
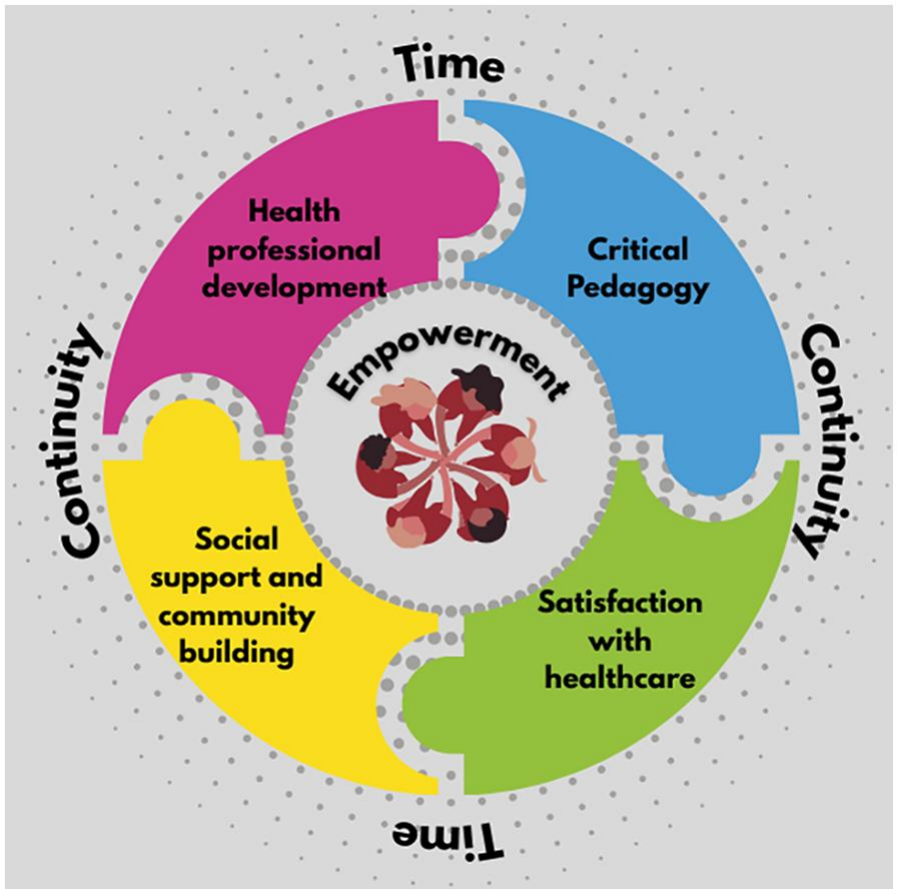
Infographic of group antenatal care mechanisms and their supporting elements.

A framework of mechanisms theorised in the literature (18) was adapted and developed more fully through this analysis. The mechanisms were closely interlinked, with time and relational continuity emerging as foundational; the longer sessions allowed for an interactive approach with the potential for deeper connections and learning. Equally, the continuity of facilitators and group participants was key to enabling the group to develop as a “safe space,” enhancing trust and allowing concerns or worries to be discussed openly and for clinical checks to take place in the group space without overwhelming privacy concerns. Continuity enabled peer support to build and learning to be reinforced, and this applied to the professionals in the space and the participants. Midwives already working in continuity models were able to integrate the group approach, observing that interaction in the groups and the longer visits enhanced their understanding of the women's needs. Figure 2 provides an infographic overview of these interlinkages.

The findings on continuity from the provider and user perspectives, namely, that it enables learning from experience and the growth of trust and meaningful communication that underpins informed choice, echoed those of prior studies on how continuity of carer functions in practice (27, 28, 38). The midwives in continuity models also spoke of being able to practice what they perceived to be “real” midwifery (29, 30) and the professional satisfaction of providing high-quality care, both of which were echoed in the midwives' experiences of Pregnancy Circles and in a systematic review of providers' experiences of facilitating group care (31). The findings also highlighted the ways in which a lack of continuity of facilitators could undermine the fidelity and functioning of the group care approach. A study on group antenatal care cites community building and empowerment as key benefits of the approach, but each had been relatively underdeveloped conceptually (18). Our analysis illuminated the ways in which each can be enhanced in group care. The interactive approach and self-checking element, supported by time for discussion, appeared to enable deeper learning. Jakubowski et al. found that while promoting empowerment through self-testing was widely acceptable to clinicians and patients, there can be a reluctance on both sides to move away from the “clinical gaze” (32). In our study, this move was a gradual, negotiated transition: the women gained confidence in their abilities at different rates, facilitated by the midwives' oversight and support. In turn, the midwives needed to witness the women's capacity before they could “relax” out of their surveillance role. Jakubowski et al. suggest that self-testing can be both disruptive to traditional hierarchies and an intensification of surveillance. Arguably, the intimacy of the Circles could be construed as an extension of surveillance, enhancing disclosure and thus the clinicians' reach into the women's lives. Nevertheless, the women in our study described the experience of participatory surveillance as empowering, increasing their confidence in seeking information and decision-making. We introduced the concept of critical pedagogy since this deeper learning was also associated with empowerment, suggesting a more transformative approach than traditional health education. The formation of peer support for many of the participants was not dependent on similar social characteristics so much as their shared journey of pregnancy, with connections continuing into early parenthood in many cases. The study period was not sufficient to learn how enduring such connections may be or whether these may translate into an enhanced capacity to gain social support from others and build a sense of community.

While the concept of critical pedagogy entails a transformative, power-shifting intention, further study is needed to explore how far this approach to care is able to achieve a transformative effect, particularly considering that in most settings it does not extend into the intrapartum period. In addition, our analysis of implementation experiences (26) highlighted how structural influences in the wider organisation, maternity system, or indeed social system may limit this potential. The current pilot work on the implementation of a Pregnancy and Parenting Circles approach in an integrated care system may elucidate this question. Our analysis highlighted that, in general, diversity in group care was experienced positively and was observed to encourage more active questioning and learning and peer support, but we also identified cases where the participants felt different from their peers and expressed concerns about being able to broach uncomfortable topics or had fears of stigma. While this is known to be a problem in individual antenatal care, our observations highlighted areas where the group facilitation skills of midwives, including sensitive conversations, needed development. The peer review sessions offered to all the participating midwives following their training workshops were rarely attended, which in some cases reflected a lack of perceived need, but more often was due to a lack of time allocated to staff reflection or development.

Perinatal peer support is known to improve psychosocial outcomes in pregnancy and may have benefits for those providing and those receiving support (33). Anthropologists use the term “biosociality” (34) to describe how groups can be transformative for people linked by a biological issue (in this case, pregnancy). Active peer support in groups can be a powerful tool to combat isolation and build a sense of community, but the biosocial environment, as we found, can also cause individuals to feel excluded, requiring attention and maintenance to bring people together (34). In a trial involving a social support intervention during pregnancy, Oakley et al. did not find a significant increase in the primary outcome measure of birthweight but noted that the participants had obtained more support postnatally than those in the control group (35, 36). The Pregnancy Circles trial found a non-significant trend towards higher social support, and, although both groups reported lower social support postnatally, this was higher in the Circles group (26). We also found that fidelity in terms of the midwives' skills and confidence in using a facilitative approach was important, and this was underpinned by continuity. For a few individuals, the sense of social support and feelings of trust that would have enabled them to share worries or concerns were not present, particularly if the group was small and lacked consistency. Moreover, a number of trial participants did not receive group care throughout the trial as a result of the COVID-19 pandemic restrictions. The findings highlight the importance of tailored training and support to consolidate the skills of those facilitating the groups, as well as the potential for a change in approach in pre-registration education to develop group care skills.

The findings highlighted that the midwives and participants found the group approach to be empowering. This was also supported by the significant increase in Pregnancy Related Empowerment Scale and health literacy scores (26). In addition to direct references to empowerment, this mechanism was supported in the way the women described how the self-checking and interactive discussions built greater confidence and understanding. Nieuwenhuijze and Leahy-Warren, in a concept analysis of empowerment during pregnancy and childbirth, highlighted external and internal attributes. External attributes are conditions that influence and may constrain or facilitate internal attributes. Internal attributes include a sense of control, self-efficacy, and belief in one’s own ability to achieve meaningful goals (37). A further aspect identified in our study was the empowerment of the midwife participants, who felt that working together, continuity within the group, and developing their facilitation skills built their own capacity to offer high-quality midwifery care. This rested on the midwives having timely and appropriate training and support, including scheduling and autonomy to ensure group continuity. Importantly, empowerment was not viewed as a “zero-sum game”, but rather as aligned with a critical pedagogy where learning was mutually constitutive and transformative (24, 25).

## Strengths and limitations

A strength of this study was the inclusion of a range of data sources and perspectives, including observations of care, focus groups and interviews with a range of participants, and reviews of meeting notes, reflections, and workshop evaluations. The thematic findings from the qualitative data were also compared with free-text survey comments from a much wider sample of participants, with consistent overall findings. A key limitation was the inability to interview those who declined to participate in the study, but we were able to interview a proportion of those who withdrew from Circles. The study period did not allow for longer-term follow-up, and this is an area recommended for future work. The potential impact of Circles on birth partners/fathers was underexplored and would benefit from further research.

## Conclusions

The theorised mechanisms from our prior realist review of group antenatal care were supported by our study’s findings, which provided further depth and detail, particularly with respect to the empowerment and learning of the facilitators and participants. The mechanisms were found to be mutually constitutive, with continuity and time forming key pillars supporting them. These aspects have not been highlighted in previous studies on group care. On these foundations, the facilitative and interactive approach fostered deeper learning and growth of trust and self-confidence. We have described this approach as a critical pedagogy since it was associated with the participants feeling a greater sense of empowerment. Together with peer support, this showed the potential for community building and improvements in wellbeing beyond pregnancy, but longer-term research is needed to explore this fully. An analysis of the integration of group care into continuity midwifery models and further work on how best to increase participation in diverse groups, including midwifery skills, are warranted. While most of the midwives responded positively to their experience of group care in this NHS setting where midwifery-led care is the norm, the degree of adaptation required was considerable and future studies on their longer-term experiences while working in more established models would be of value. The findings highlighted the importance of training and mentoring support to facilitate this adaptation, but we also found that empowerment was mutually constitutive—the midwives involved also felt a greater sense of professional satisfaction, empowerment, and even joy in their work when participating in this approach to care.

## Data Availability

The datasets presented in this article are not readily available because we did not collect any of the kinds of data that are required to be made available. Requests to access the datasets should be directed to angela.harden@citystgeorges.ac.uk.
